# New perspective of ceria nanodots for precise tumor therapy via oxidative stress pathway

**DOI:** 10.1016/j.heliyon.2022.e10370

**Published:** 2022-08-23

**Authors:** Hui Wang, Qi Wang, Jianyue Dong, Weiwei Jiang, Linghong Kong, Qiong Zhang, Hanping Liu

**Affiliations:** a MOE Key Laboratory of Laser Life Science & Institute of Laser Life Science, College of Biophotonics, South China Normal University, Guangzhou 510631, China; b Guangzhou Key Laboratory of Spectral Analysis and Functional Probes, College of Biophotonics, South China Normal University, Guangzhou 510631, China

**Keywords:** Ceria nanodots (CNDs), Reactive oxygen species (ROS), Hydroxyl radicals (OH), Tumor recognition, Fenton reaction

## Abstract

Ceria-based nanomaterials have aroused major attentions among the biomedical application research field in recent years. Most of the researches have mainly focused on promoting the functional healing therapies of normal cells/organs with cerium oxide compounds, while the applications of ceria-based materials employed on cancer curing processes have been merely mentioned. To explore the possible capabilities of cerium oxide nanomaterials exterminating tumor cells, innovatively, we synthesized the eco-friendly pure cerium oxide nanodots (CNDs), proving the prominent ability of CNDs used in tumor chemotherapy (CDT) via Fenton reaction with the highly presence of H_2_O_2_ (acidic pH) in tumor tissues. CNDs reacted with the self-produced H_2_O_2_ of tumor cells, which generated piled up toxic hydroxyl radical (·OH). The accumulated virulent ·OH restrained the growth of cancer cells intensively. This peroxidase-like activity, provided a distinguished paradigm for effective cancer curing treatment. We also verified the biosafety of CNDs applied on normal cells. Notably, not only did CNDs be harmless to normal cells, but also it protected them from the damages of reactive oxygen species (ROS). In normal cells/tissues, under the microenvironment of neutral pH and low level of H_2_O_2_, the CNDs could effectively function as an annihilator inhibiting ROS. They reduced the damages caused by ROS, exhibiting catalase-like activity. The research we studied, which estimated CNDs thoroughly, has provided a new perspective to the future researches of the cerium oxide biomaterial applications.

## 1 Introduction

Cancer, one of the leading causes of lethality, still remains a series of frustrating therapeutic conundrums over the past decades. The uncontrolled fragmentations and proliferations of cancer cells are the main features as well as the predicaments of the cancer healing process [1, 2]. What cannot be neglected is that the weak selectivity of the cures, which somehow takes the main responsibility of multidrug resistance [3]. To solve the problem mentioned above, most commonly, researchers choose to utilize the particular biological mechanism to design the experiments that might be advantageous to the recovery of cancer sufferers [4, 5, 6].

**Scheme 1:**
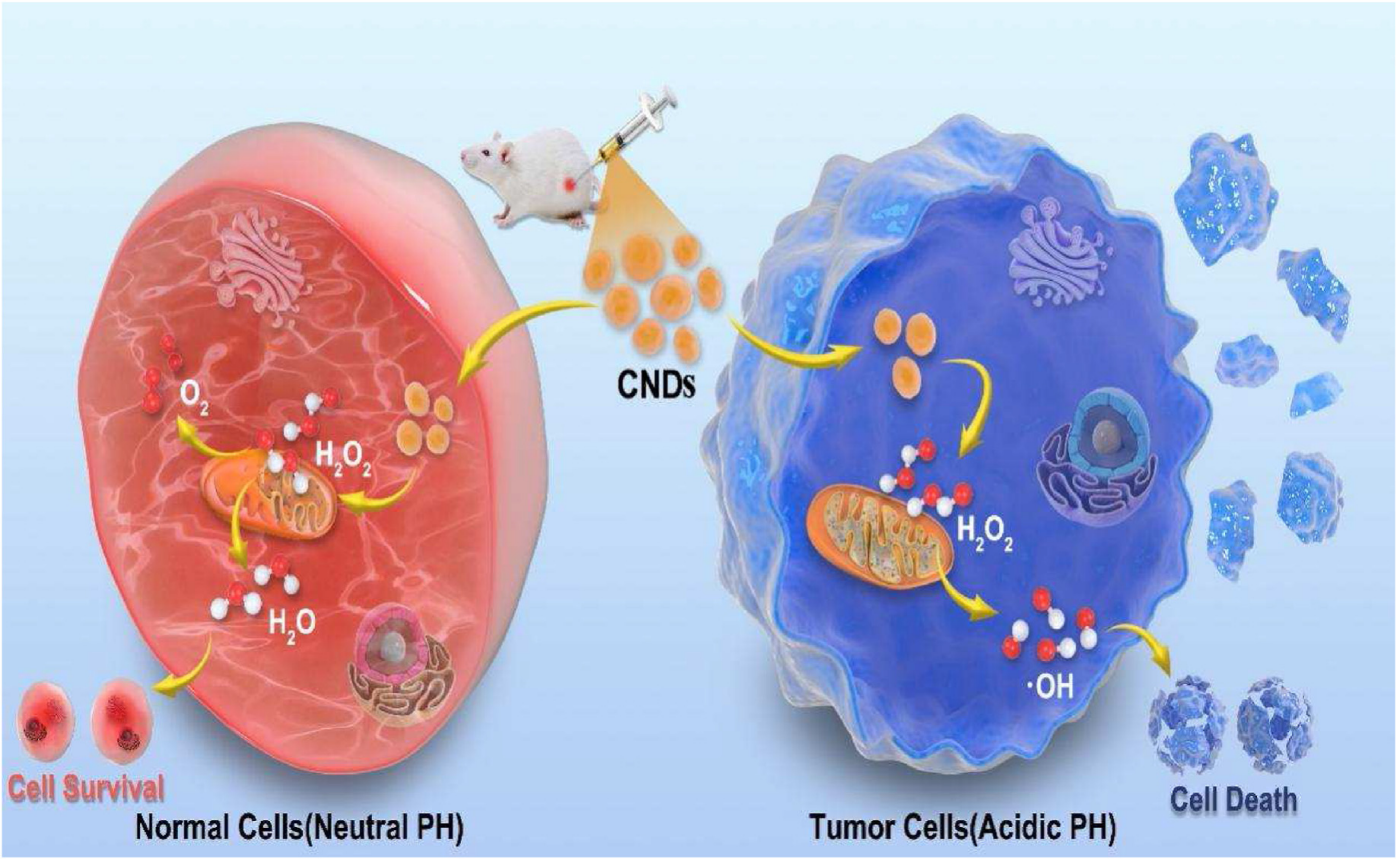
Schematic illustration of CNDs reacting with ROS in normal cells and cancer cells.

Especially, the amount of research outputs related to glucose metabolism in cancer cells was remarkable [7, 8, 9, 10]. Known as the “Warburg effect”, in cancer cells, ATP would still more likely to be generated through glycolysis, even under the circumstance of owing enough oxygen. With the operation of glycolysis, on the contrast of other cells, more H^+^ would be produced around the tumor cells, causing the acid environment in the surrounding of cancer cells [11, 12, 13, 14, 15]. The existence of abundant hydrogen peroxide is also a significant feature of tumor microenvironment. Although, specific reason of why H_2_O_2_ accumulated around cancer cells still remains mysterious [16]. The characteristics of hydrogen peroxide’s accumulation among tumor cells’ surrounding environment have been reported continuously over the past decades [17].

On the basis of such exceptional distinction, certain drug delivery system could be drafted up in order to target on cancer cells. CDT, as an unconventional strategy for traditional cancer treatment, has attracted increasing attentions of researchers in recent years due to its unique way of generating ROS [18, 19, 20]. The presence of ROS can cause damages to mitochondria and DNA, bringing the cells cycle to standstills [21, 22]. On the one hand, this does cause considerable damages to normal cells, and certain methods should be devised to reduce the amount of reactive oxygen species in normal cells. On the other hand, we can utilize this feature to enable tumor cells to produce a certain amount of reactive oxygen species, resulting in the death of tumor cells [23, 24]. In recent years, the rise of nanoparticles has brought numerous researchers devoted into the studies of using nano-components to cure cancer via CDT. Thereinto, Fenton reaction is a worth discussing therapeutic approach. ·OH can be generated directly by endogenous chemical energy (H_2_O_2_) through Fenton reaction mediated by metal ions, causing pernicious oxidative damages to cancer cells [25, 26]. Researchers have explored the meditation effects involved Fe, Cu, Mn and so on [27, 28, 29]. However, the safety of certain metal nanoparticles and the difficulties of synthesis remain huge spaces for the discussions of the oncology treatment.

Under the circumstances mentioned, rare metal oxides stepped into our sight [30]. Particularly, the attentions on ceria components emerged in increasingly. The unique regulated variation of valence between Ce^3+^ and Ce^4+^ invest ceria with the ability of accommodated the balance of ROS among regular cells dynamically [31, 32, 33]. Cerium oxides have been used in several diseases’ recoveries so far, such as inflammation [33], chronic wound healing [34], Alzheimer's disease [35, 36] and so on.

Most of the cerium-based drug systems have the sizes of 10–200 nm [36, 37]. However, according to studies on nanomaterials such as cerium-based materials by different research teams, it was found that sub-5 nm nanomaterials have better surface-area-to-volume ratios and can provide more sites for reactive oxygen species reactions, allowing the reactions to proceed more efficiently [35, 38]. In addition, nanomaterials with smaller sizes have better vascular permeability. In tumor cells, nanoparticles that enter the tissue will be retained in the tumor cells due to the Enhanced Permeability and Retention (EPR) effect. In detail, due to the significantly higher growth rate of tumors than normal tissues, the defects between tumor vascular endothelial cells, which are not closely aligned and permeable, and the insufficient lymphatic drainage within the tumor and low blood flow rate, nanoparticles will be retained in the tumor site once they enter [39]. This is one of the reasons why the sub-5 nm material has stronger tumor targeting effect.

Recently, scientists have noticed that ceria might be advantageous in the process of cancer healing. The studies of the functions of combined ceria and other materials acting on tumor curing have been conducted by few research teams [37]. However, the discussions were mainly focused on the assisted capacity of ceria being as one of the auxiliary components of the drug delivery systems [40]. The mechanism of ceria alone performing in clearing cancer cells was barely discussed by the researchers. The effects of using cerium oxides only, during the procedure of killing tumor cells or even curing the cancer suffered animals while protecting the normal cells from being damaged, had merely been mentioned on the previous studies of cerium oxides related researches.

Here, For the first time, we synthesized the eco-friendly as well as maneuverable ceria nanodots (CNDs). By establishing a series of in vitro/vivo experiments, we have proved that the CNDs we synthesized could be capable of targeting on tumor cells and complete the Fenton like reaction with H_2_O_2_ to kill the tumor cells under the acid tumor microenvironment causing by the “Warburg effect”, as shown in Scheme 1. Excessive amount of ·OH, which could trigger the necrosis of tumor cells, would be produced after the peroxidase-like activity causing by CNDs. Followed up, Cancer cells would be killed, due to the piled up highly toxic·OH. By contrast, under the neutral environment with normal volume of H_2_O_2_, the regular cells would continue with the general metabolism function. Moreover, the CNDs, to a certain extent, could protect the normal cells from the damages of exogenous ROS.

## 2 Materials and methods

### 2.1 Preparation of CNDs

Briefly, 0.25 g cerium acetate (Macklin, China) was gently added into 15 ml of deionized water preheated at 35 °C, stirred vigorously at constant temperature (35 °C). Next, drop by drop, acetic acid (XiLong Scientific, China) was added to the solution to adjust pH to 6.0. Continue to stir at constant temperature for 6 h. After stirring, the solution was dried at 50 degrees centigrade under vacuum for 24 h. Finally, the recovered sample powder was washed with deionized water for three times.

### 2.2 Characterization of CNDs

The TEM images of CNDs solution with a concentration of 0.35 mM were obtained with a JEM-2100HR transmission electron microscope (JEOL, Japan). The CNDs (0.02 g) were fully reacted with H_2_O_2_ (1.0 mM), and the supernatant was discarded when no more bubbles were generated, dried under vacuum and washed three times with deionized water. The reacted sample (0.01 g) and the original sample (0.01 g) were analyzed by XPS separately. XPS spectra were obtained with a XPS spectrometer (ThermoFisher Nexsa, America). The Raman spectra of CNDs and CNDs + H_2_O_2_ were recorded by 488 nm laser. Raman spectra were obtained with a Alpha300R Raman spectrometer (Wealtec, Germany).

### 2.3 Ultraviolet−visible−near-infrared (UV−vis−NIR) absorption spectra of CNDs

Different concentrations of CNDs were reacted with different concentrations of H_2_O_2_. Ultraviolet−visible−near-infrared (UV−vis−NIR) absorption spectra of the solutions were obtained using a UV−vis−NIR spectrometer (Shimadzu, Japan). TMB was added to different solutions (CNDs, H_2_O_2_, CNDs + H_2_O_2_) and the UV−vis−NIR absorption spectra of the solutions were measured at 650 nm.

### 2.4 ESR spectra of CNDs + H_2_O_2_

H_2_O_2_ was added to the CNDs solution. After the completion of the reaction, trapping agent, DMPO, was added to capture the hydroxyl radicals. Electron spin resonance (ESR) spectrum of the solution was obtained with an A300 ESR spectrometer (Bruker, Germany).

### 2.5 Cell culture

HeLa cell lines were cultivated in DMEM with pH of 6.0 (iCell Bioscience, China) and 7.4 (Gibco, America) separately, containing 10% FBS (Gibco, America), 1% antibiotics (Gibco, America) at 37 °C in a humidified atmosphere with 5% CO_2_. DMEM with neutral pH, containing 10% FBS and 1% antibiotics was used to culture the Mesenchymal Stem Cell lines. The cultivation was proceeded at 37 °C in a humidified atmosphere with 5% CO_2_.

### 2.6 In vitro cytotoxicity of CNDs

HeLa cell lines were added to 96-well plates at a density of 3000 cells per well. After incubating for 12 h, the mediums were discarded. Followed up, Rinsing the cells twice with phosphate-buffered saline (PBS, pH 6.0). The CNDs solution was added separately according to the concentration gradient (0.5 mM, 1.5 mM, 2.5 mM, 3.5 mM, 4.5 mM, 5.5 mM). Acidic DMEM medium was also added. After 12 h of incubation at 37 °C, medium was discarded again, and the cells were washed three times with PBS (pH 6.0). Using a Kit-8 (CCK-8 assay, Beyotime, China), 100 μL per well, containing DMEM medium to obtain the cells count. Finally, the microplate reader was used to read the counts after 2 h of incubation.

### 2.7 Observation of ROS

HeLa cells (pH 6.0)/Mesenchymal Stem Cells (MSCs) were added to 6-well plates at a density of 1 × 10^5^ cells per well separately for 12 h. Then, the mediums were discarded. Followed up, cells were washed three times with PBS, CNDs solution (5.5 mM)/CNDs solution (5.5 mM) + H_2_O_2_ (1 mM)/CNDs solution (5.5 mM) + PBS with DMEM was added into each well. After cultivating for 24 h, the fluorescence probe 2′,7′-dichlorofluorescin diacetate (DCFH-DA, Beyotime, China) was added to each well. Observing the fluorescence substance (λex = 480 nm, λem = 525 nm) under a confocal microscope in 15 min.

### 2.8 Observations of viable and dead cells

After culturing the HeLa cells (pH 6.0, pH 7.4) and MSCs in φ 15 CLSM-exclusive culture disks for 12 h, CNDs were added to each disk. The cells were cultured for 24 h, stained with calcein (AM, Beyotime, China)/propidium iodide (PI, Beyotime, China) and then incubated for 20 min. Confocal fluorescence spectra were recorded by 480 nm and 545 nm lasers.

### 2.9 Tumor mouse model

All female BALB/c nude mice were offered by Southern Medical University in Guangdong Province under the Animal Protection and Utilization Committee of the South China Normal University. After the mice aged 6 weeks, 4 × 10^6^ HeLa cells which were suspended in 200 μL of serum-free DMEM (containing 1% pen/strep, 100 U/mL penicillin, and 100 μg/mL streptomycin) were injected subcutaneously into their thighs. When the tumor volume reached 200 mm^3^, the mice were divided into two groups (normal saline/CNDs-treated, n = 4) and photographed with digital camera.

### 2.10 Intratumoral therapy in tumor mouse model

Normal saline (200 μl)/CNDs solution (200 μl) were injected intratumorally into the mice separately. The volumes of tumors were measured with digital Vernier calipers once two days. On day 9, the pictures of mice were also documented by photographs. Followed up, all the mice were sacrificed. The major organs and tumors were dissected, collected, and used for Histological examination after sectioning into thin slices (10 μm). Histological examination of tumors and main organs were taken by hematoxylin and eosin (H&E) staining.

### 2.11 Toxicology experiment in vivo

BALB/c mice were divided into two groups (normal saline/CNDs-treated, n = 4). CNDs were injected intravenously into the mice separately. 3 days later, blood was collected from each group of mice by orbital sinus blood sampling for routine blood tests. Routine blood examinations were monitored to certify the safety of CNDs.

### 2.12 Statistical analysis

All data are expressed as the mean difference ±standard deviation. Differences between experimental groups were analyzed using Student’s t-test. The Pearson correlation coefficient was calculated to estimate the linear correlation between two variables. Statistical analysis was performed using Origin 2019b (OriginLab, America). All tests were 2-tailed, and *P* < 0 .05 was considered statistically significant.

## 3 Results

### 3.1 Characterization of CNDs

**Figure 1:**
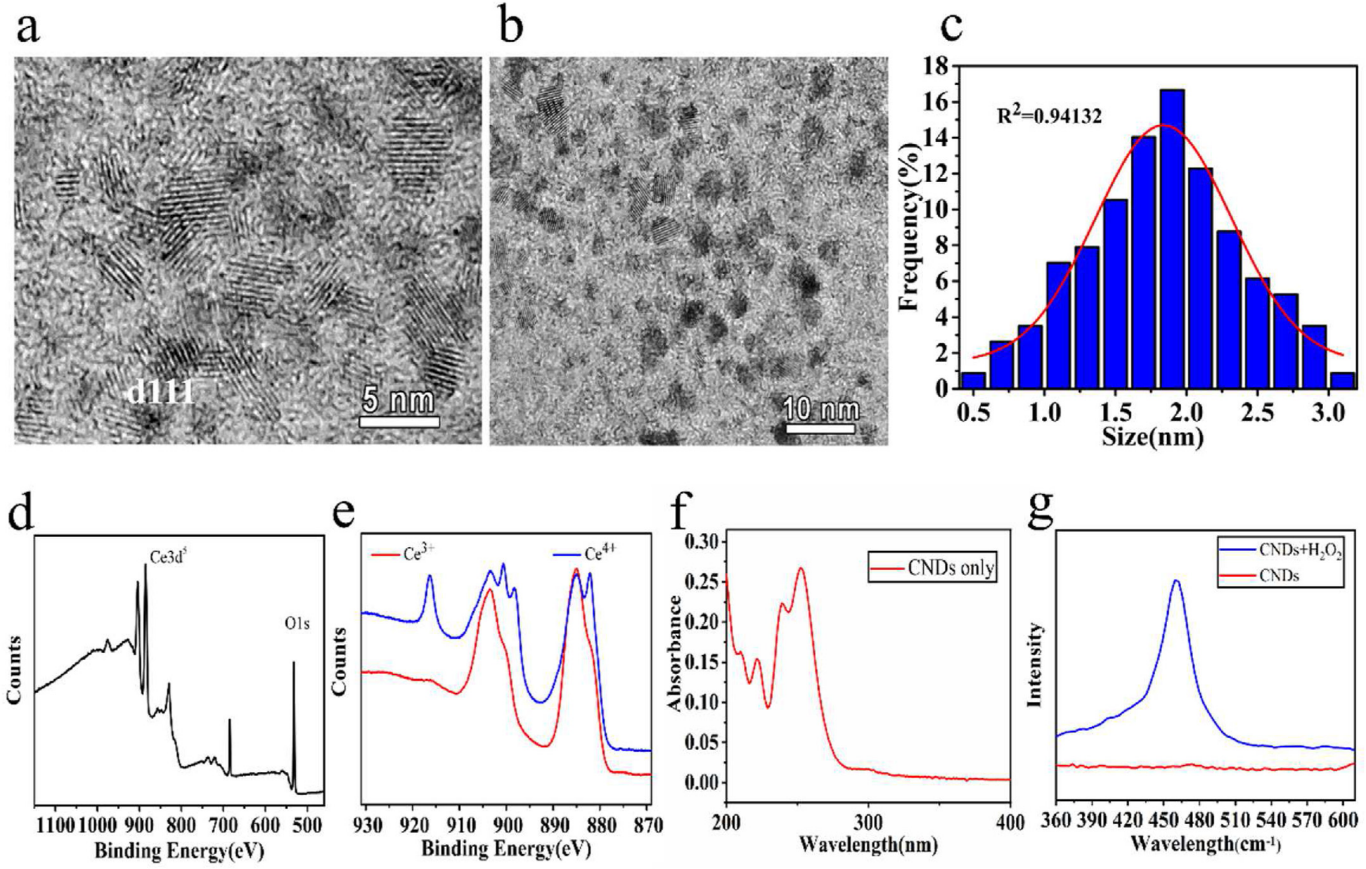
(a, b) HR-TEM images of CNDs. (c) Particle diameter distribution chart of CNDs. (d) XPS analysis of CNDs. (e) XPS analysis of valence state of Ce before/after reacting with H_2_O_2_. (f) UV-vis absorption spectra of CNDs dispersed in water before reacting with H_2_O_2_. (g) Raman spectra of CNDs before/after reacting with H_2_O_2_.

**Figure 2:**
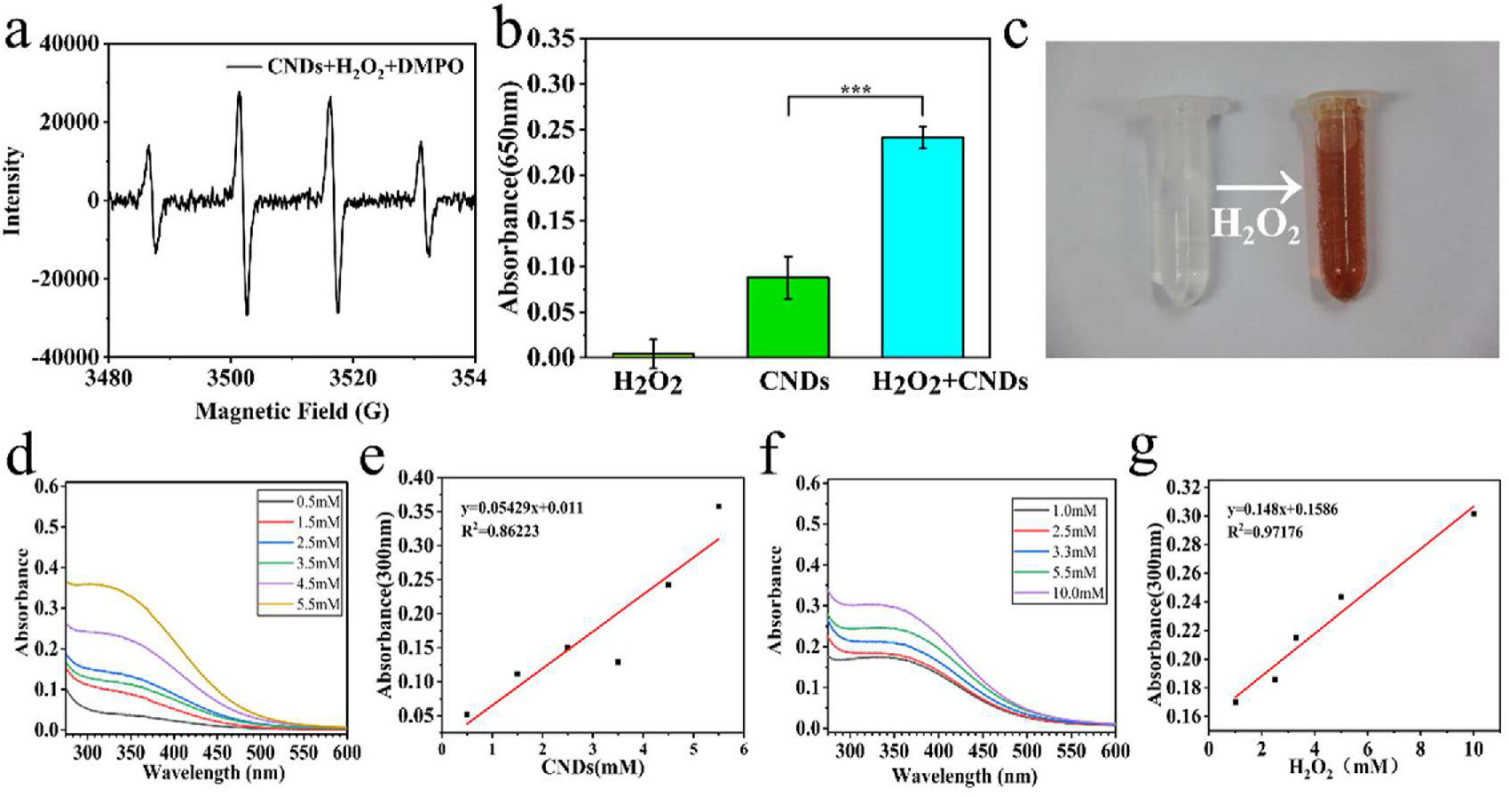
(a) Spin-trapped ESR spectra of OH captured after the reaction system involved CNDs, H_2_O_2_, DMPO. (b) UV-vis absorptive intensity of H_2_O_2_, CNDs, H_2_O_2_+CNDs at 650nm. (∗∗∗*P* ＜ 0.001). (c) Aqueous solutions of CNDs reacting with H_2_O_2_ before/after. (d) UV-vis spectra of gradient concentrations of CNDs reacting with H_2_O_2_ (1 mM). (e) Linger relation of different CNDs concentrations and H_2_O_2_ at 300 nm of UV-vis. (f) UV-vis spectra of CNDs (5 mM) reacting with gradient concentrations of H_2_O_2_. (g) Linger relation of CNDs and different H_2_O_2_ concentrations at 300nm of UV-vis.

The size and morphology of CNDs were characterized by HR-TEM. As is shown on the TEM graph (Figure 1a), the CNDs were successfully synthesized with lattice fringes of d111 = 0.31 nm. The CNDs distributed in aqueous solution uniformly (Figure 1b). After analyzing the distributions and the diameters of CNDs, we concluded that the sizes of CNDs are around 2.0 nm, which also fit the normal distribution curve (Figure 1c). XPS, Raman and UV-vis were used to verify the synthesis and the alteration of valence state of CNDs before and after adding H_2_O_2_. XPS analysis shows that the nano particles were composed with Ce and O (Figure 1d). Before adding H_2_O_2_ into CNDs, presented as the red line on the graph of XPS analysis of Ce (Figure 1e), the component was mostly consisted with Ce^3+^ (peaks at 885.80 and 902.80) [41]. The 5d energy state of Ce^3+^ split into 3 substates, which exhibited on UV-vis spectrum of CNDs (Figure 1f) are peaks at 221 nm, 240 nm and 254 nm. After reacting with H_2_O_2_ (Figure 1e), part of the Ce^3+^ were oxidized to Ce^4+^ (peaks at 881.80, 900.60 and 916.50) [42]. Broad absorption bands could also be observed in the region between 320 nm and 450 nm (Figure 2d), corresponding to the charge transfer leap from Ce^4+^ to O^2-^ that occurs in CeO_2_ [43]. The Raman spectrum (Figure 1g) displays a strong characteristic peak of CeO_2_ at 462 nm [44], after the reaction of CNDs and H_2_O_2_. Before the reaction with H_2_O_2_, the CNDs did not have the corresponding Ce^4+^ Raman peak, from which it could be concluded that the content of CeO_2_ in the CNDs was extremely low before the reaction (Figure 1g). To conclude the above experiments, the CNDs have been successfully synthesized in an environmentally friendly way, after reacting with oxidizing agent, the CNDs which are Ce_2_O_3_ dominated would mostly change into CeO_2_.

### 3.2 The fenton-like reaction of CND in vitro

Ce_2_O_3_ + 2H_2_O_2_ +H^+^ = 2CeO_2_ + ·OH + 2H_2_O

Under the environment of the presence of excessive amount of H_2_O_2_, CNDs would complete the Fenton like reaction, producing ultra hydroxyl free radical [26]. We used 5,5-dimethyl-1-pyrroline N-oxide (DMPO) to capture the ·OH (Figure 2a), the electron spin resonance (ESR) was applied to identify the capability of the reaction producing ·OH. An intense characteristic ·OH/DMPO peak (1:2:2:1) was observed after adding H_2_O_2_ (1.0 mM) into the CNDs solution [24], The pH of the CNDs solution for all experiments was 6.0, and the acidity of the solution was set to simulate the acidic environment of tumor tissue [20, 29]. The characteristic absorption peak of the reaction system, including CNDs (5.5 mM), H_2_O_2_ (1.0 mM)and chromogenic 3,3′,5,5′-tetramethylbenzidine (TMB) could be observed at 650 nm. By contrast, the absorption peaks of the other two solutions were neglectable. The choice of CNDs concentration was determined by cytotoxicity assays in this study, and the choice of H_2_O_2_ concentration was determined by the hydrogen peroxide concentration of the tumor microenvironment [45]. Within the realm of statistics, the result of CNDs + H_2_O_2_+TMB has significant difference (*P* < 0.01) comparing with CNDs only (Figure 2b), which indicates that the peroxidase-like catalytic activity happened after adding H_2_O_2_ (1.0 mM) into CNDs solution (5.5 mM). We can witness the reaction visually by observing the rapid color change of the solution (Figure 2c). The CNDs solution changed from colorless to deep brown when we added H_2_O_2_ in it. The complete degree of the reaction is related to the concentration level of the H_2_O_2_ and CNDs. The absorption characteristic appears to be decreasing along with the reduction of the gradient change of H_2_O_2_. Also, with the descending of the concentration of CNDs, the absorbance reduction could be noticed (Figure 2d). The concentration alteration had linear relationship with the reaction degree (Figure 2e). The intensity of the reaction is also related with the concentration of H_2_O_2_. With higher concentration of H_2_O_2_, the reaction would be more complete (Figure 2f), which is also linearly dependent (Figure 2g). Combining the above results to conclude, in an acidic environment, H_2_O_2_ added with CNDs generated substantial amount of ·OH immediately, trapped by DMPO [26]. After the Fenton like reaction, the released OH oxidized the colorless TMB, causing an absorption peak spotted at 650 nm.

### 3.3 Treatment effects of CNDs in vitro

Among the metabolisms of cells’ biochemical processes, H_2_O_2_ would be produced in a certain amount, causing a series of ROS related reactions, which would be damaged to cells. Due to the homeostasis of the organism itself, normally, it would not cause vital injury. However, the cumulate hydroxyl radicals would be fatally harmful to cells. To prove the nanoparticles’ curative effect towards cancer cells, we have established and implemented several experiments in cellula dimension to prove the efficiency of the CNDs.

**Figure 3:**
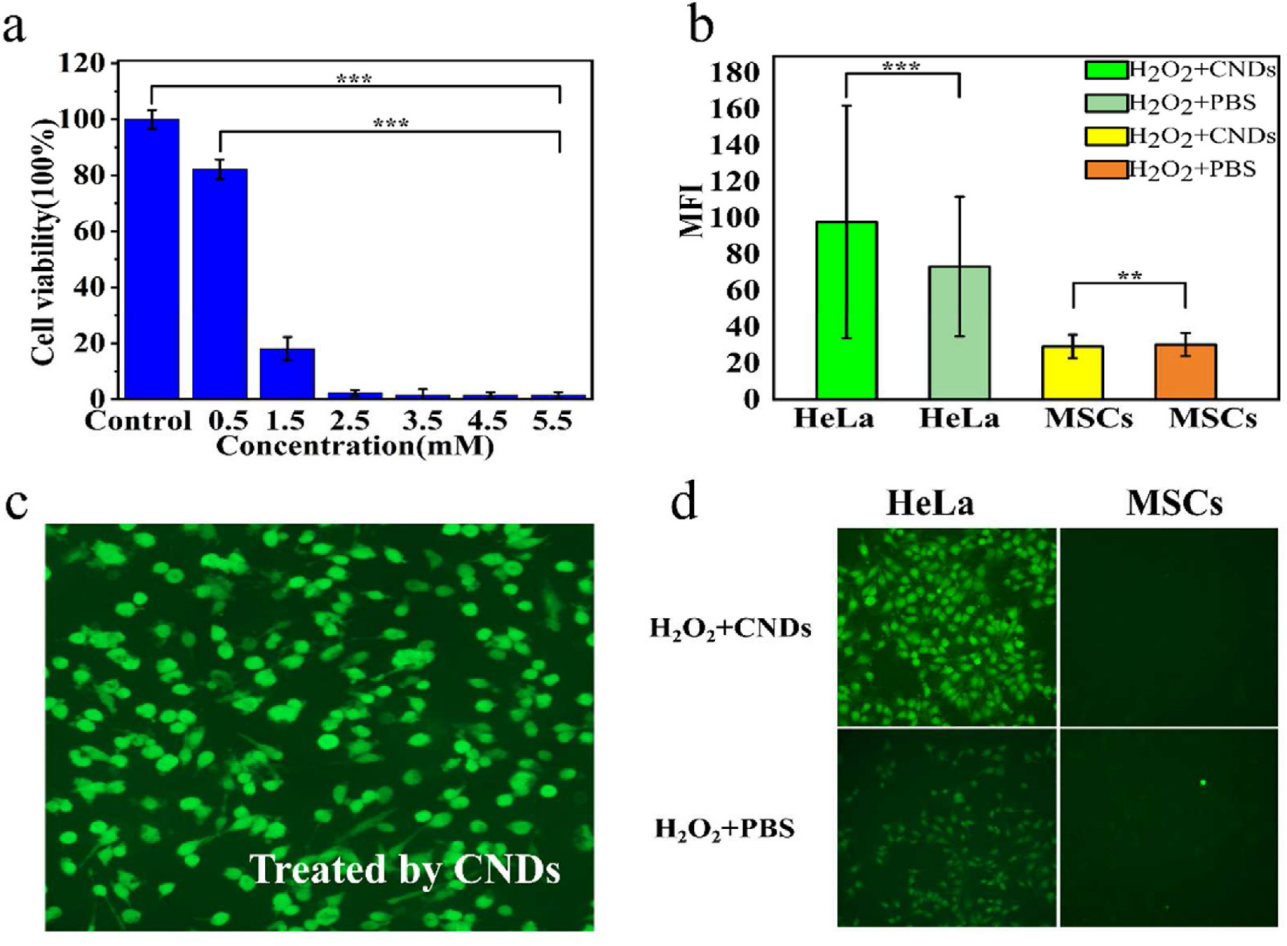
(a) In vitro HeLa tumor cells cytotoxicity profiles for CNDs (pH 6.0,∗∗∗*P* ＜ 0.001). (b) MFI values of H_2_O_2_ treated HeLa cells and MSCs (∗∗*P* ＜ 0.01, ∗∗∗*P* ＜ 0.001). (c) Intracelluar ROS detection after HeLa cells treatment with CNDs (5 mM). (d) Intracelluar ROS detections after HeLa cells and MSCs treated with H_2_O_2_+CNDs/PBS.

In order to monitor the cytotoxicity of CNDs, and determine the appropriate reaction concentration. HeLa cells were cultivated with different concentrations of solutions separately for 24 h, in the order of certain concentration gradients (0.5 mM, 1.5 mM, 2.5 mM, 3.5 mM, 4.5 mM, 5.5 mM). The experiments of toxicity were carried out by using the Cell Counting Kit-8 (cck-8) assay. We can learn from the chart that the vitality of cells highly depends on the concentration of CNDs solutions (Figure 3a). The viability of tumor cells depends on the concentration of the cerium oxide nanoparticles. After 24 h of medical treatment, most of the cells which was treated with 0.5 mM nanoparticles solution stay survival, with the 80% of cell viability averagely. The downtrend of cell viability along with the concentration uptrend indicates that with the increase of the concentration of CNDs solution, the effect of the nanoparticles increased prominently. When reaching 5.5 mM, the tumor cells basically all died, verifying the ability of CNDs using to damage cancer cells. Hence, in all the following cellular assays, we selected a solution of CNDs at a concentration of 5.5 mM to be studied.

To observe the ROS producing conditions in cancer cells and normal cells, we established the ROS generated experiments of different cells. Knowing from Figure 3c, the cancer cells pretreated with CNDs generated considerable amount of ROS, displayed strong green fluorescence, indicated the ability of CNDs producing toxic ROS causing the death of the cancer cells. Besides treating the cancer cells with CNDs, we also added H_2_O_2_ and PBS in different cells in order to learn the efficiency of CNDs generated ROS, reacting with H_2_O_2_ (Figure 3d). The cancer cells treated with H_2_O_2_ produced certain amount of ROS while the PBS group acquired minute amount one. Otherwise, minute amount of ROS was generated in MSCs, especially in the H_2_O_2_ treated group, which could be explained by the ability of CNDs eliminated the ROS in normal cells. The Mean Fluorescence Intensity (MFI) of adding CNDs or PBS have significant different either in HeLa cells group or in MSCs group (Figure 3b). This phenomenon indicated that in normal cells, CNDs would not cause ROS. Moreover, they would function as ROS cleaning tool, protecting the cells from the damages caused by ROS generated by other biochemistry reactions in cells. The ROS experiments in cells could be explained by the previous chemistry reaction experiments. The CNDs caused Fenton-like reaction in tumor-like condition, producing hypertoxic ·OH, killing the harmful tumor cells. In normal cells, the CNDs would more likely to continue the progress of ROS eliminated, protecting the cells from the jeopardizing harm of ROS.

**Figure 4:**
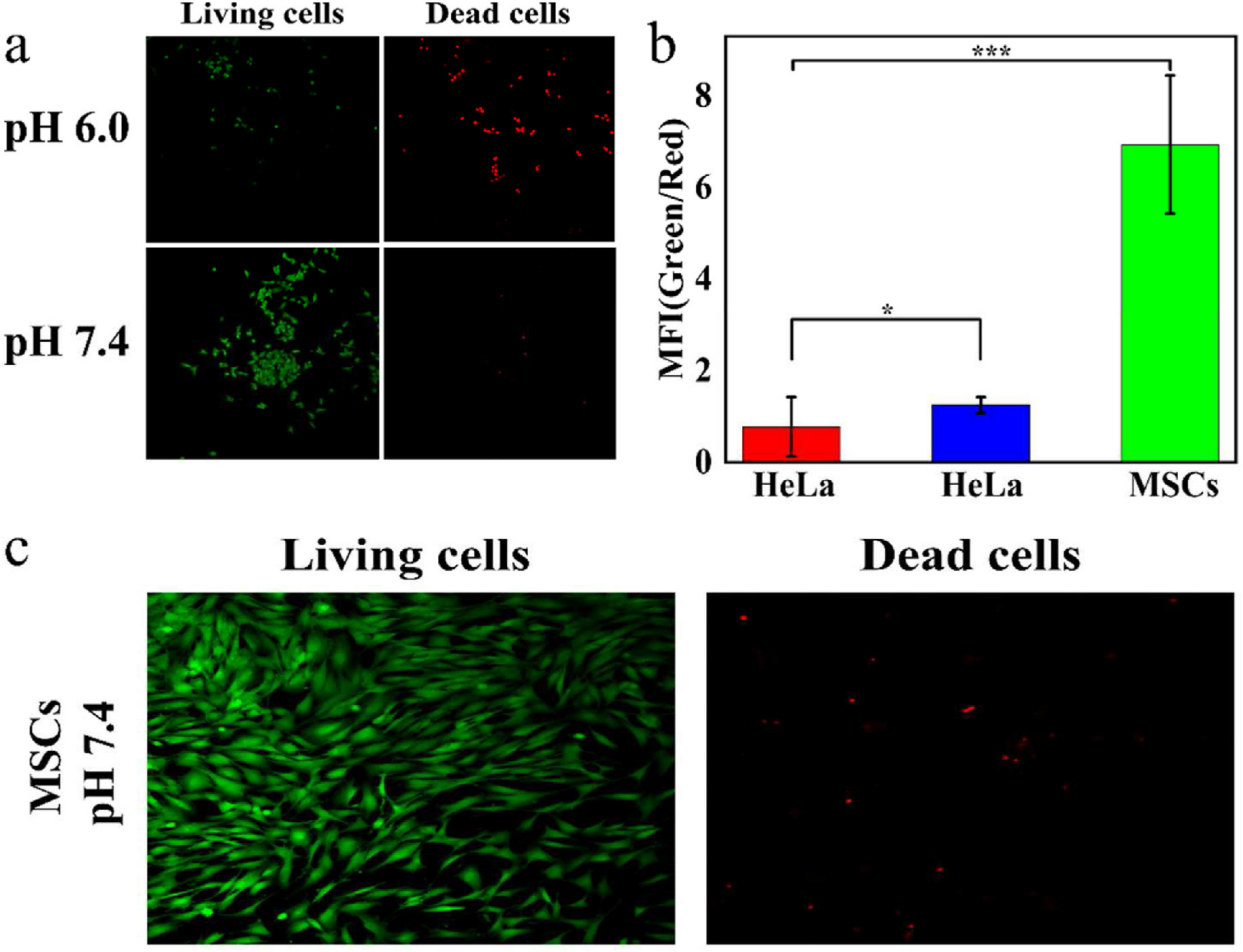
(a) Live/dead assay of HeLa cells incubated with CNDs (5 mM) under acidic or neutral pH. (b) Corresponding MFI values ratio (Green/Red) of Live/dead assay of HeLa cells (pH 6.0, 7.4) and MSCs (∗∗*P* ＜ 0.01, ∗∗∗*P* ＜ 0.001). (c) Live/dead assay of MSCs incubated with CNDs (5 mM) under neutral pH.

To visualize the effect of CNDs in a more direct way, the calcein acetoxymethyl ester (calcein-AM, green fluorescence) and propidium iodide (PI, red fluorescence) were used to prove the achievements of CNDs. HeLa cells (pH 6.0 and pH 7.4) and MSCs were cultivated with CNDs (5 mM) for 6 h separately. Then using calcein-AM or PI to stain living cells as well as dead cells which generating green fluorescence or red one. The final graphs were manifested by confocal laser scanning microscopy (CLSM). Observing from Figure 4a, the HeLa cells which cultivated in acid environment had higher death rate comparing with the HeLa cells grew under neutral condition. The statistical diagram of MFI (Figure 4b) specifically explains that, comparing with pH 7.4 culture medium, the performance of killing cancer cells of CNDs is much more effective in pH 6.0 culture medium. Besides, the MSCs treated with CNDs nearly all grew in a regular condition, proving that the nanoparticles’ biosecurity for normal cells again (Figure 4c). The differences of the three groups were mainly caused by differences in ability of CNDs resolving H_2_O_2_ under different conditions. Under acid environment in cancer cells, massive H_2_O_2_ would be decomposing into OH quickly, leading the death of cancer cells. Under the ordinary environment in normal cells, the H_2_O_2_ could be used as the catalase, helping the normal cells to reduce ROS damages, performing healthily growth cultivating with CNDs.

### 3.4 Treatment effects of CNDs in vivo

**Figure 5:**
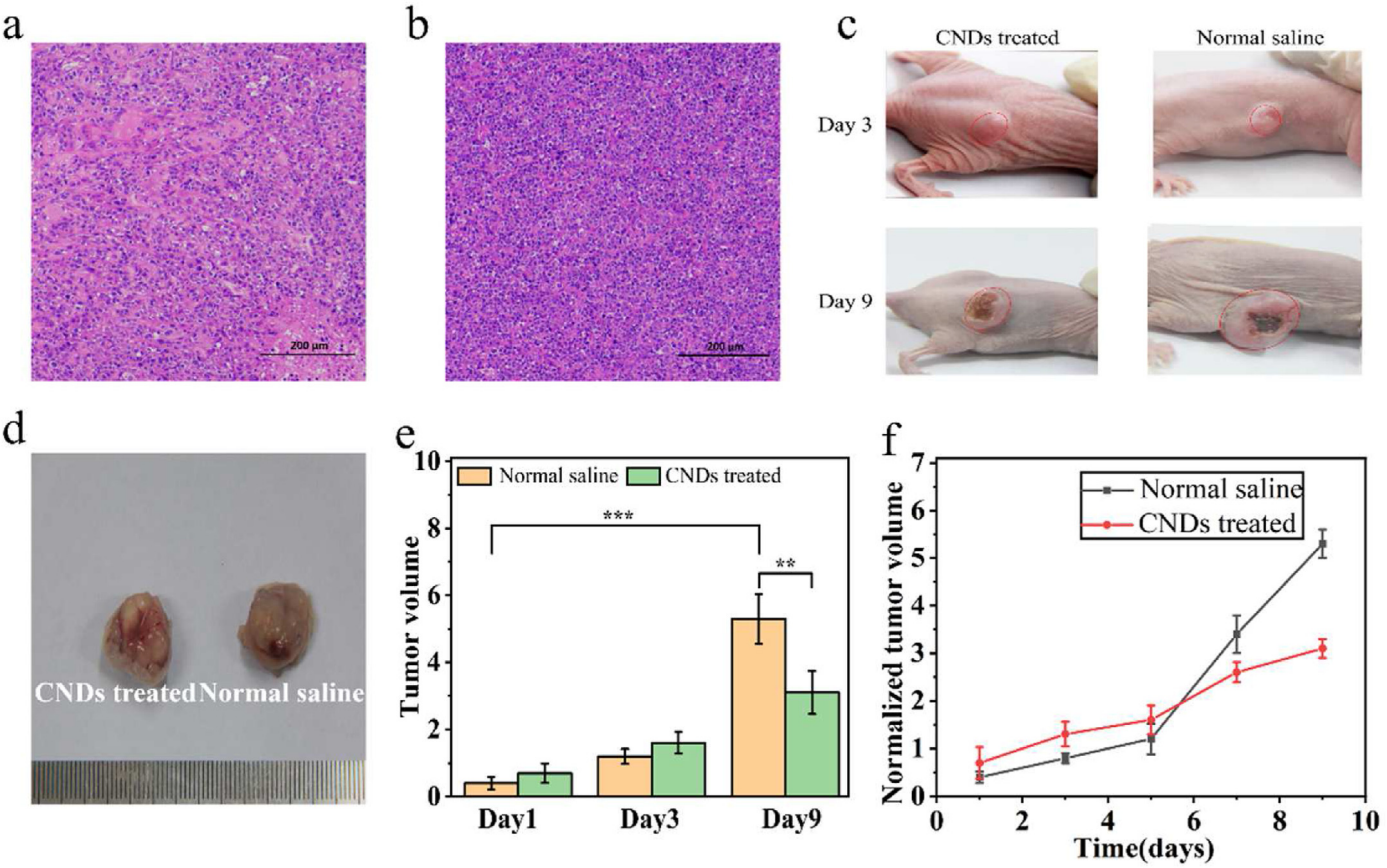
(a) Histological H&E analyses of the tumor sections from CNDs-treated group, scale bar is 200 μm. (b) Histological H&E analyses of the tumor sections from normal saline group, scale bar is 200 μm. (c) Pictures of mice of CNDs-treated/Normal saline group focused on tumor area on day 3 and day 9. (d) Representative pictures of tumors obtained from each group after anatomy. (e) The comparison of tumor volume among normal saline and CNDs-treated on day 1, day 3, day 9 (∗∗*P* ＜ 0.01, ∗∗∗*P* ＜ 0.001, n = 4) (f) Tumor growth curves of control/experiment group.

To indicate the bio-safety of CNDs in mammals, CNDs were intratumorally injected into BALB/c nude mice which were all treated with HeLa cervical tumor cells xenografts. By comparison, normal saline was injected intratumorally into the control group of mice. We monitored the growth state for 9 days after the medical therapy among the experimental group and the control group for the tests of the inhibition effects of CNDs. In order to certify the ability of CNDs decreasing the explosive generation of cancer cells, following up, we established histological experiments to verify. The graphs of control group and experimental group of hematoxylin and eosin (H&E)-stained tumor slices were taken separately. Clearly observed from the pictures, with normal saline treatment, the tumor cells maintained the original condition (Figure 5a). The membrane and nuclear were both under complete conditions in the morphology level. By contrast, the nuclear as well as the membrane status of tumor cells treated with CNDs, occurred to be distorted (Figure 5b). The comparison of the H&E-stained photographs between normal saline group and CNDs-treated group manifested the therapeutic ability of CNDs using in tumor curing progress.

The mice’s tumors condition of normal saline group as well as the CNDs-treated group were both documented by the pictures taken on day 3 and day 9 (Figure 5c). The average tumors volume of the CNDs-treated group was statistically significantly smaller than the control group’s (Figure 5e). On day 9, the volume of tumors among two groups also has significant difference (Figures 5d and 5e). The slope of growth rates curve of control group is bigger than the experimental group, which also demonstrate the conspicuous restrained effect of CNDs targeted on tumor cells (Figure 5f).

**Figure 6:**
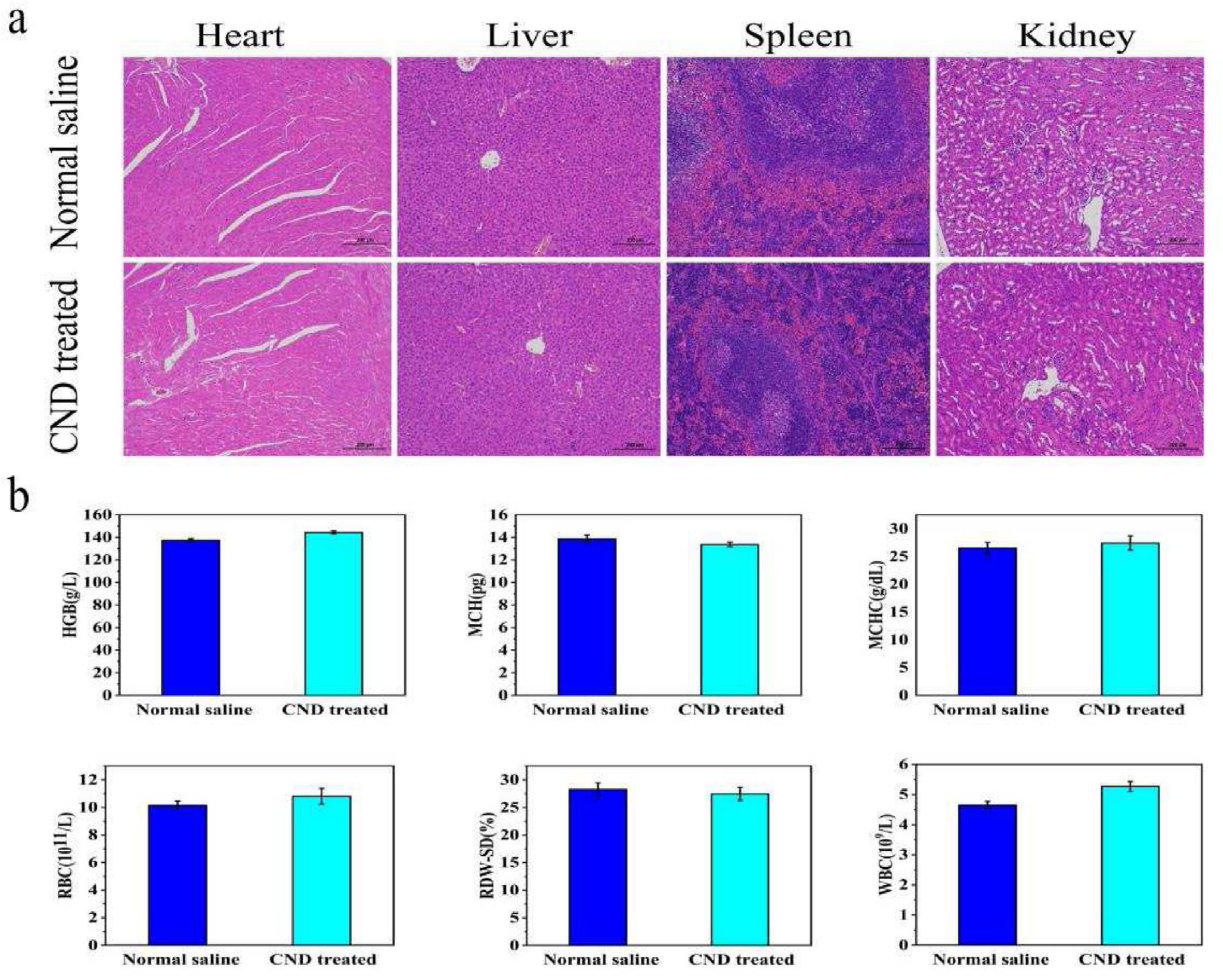
(a) H&E staining of major organs in CNDs-treated/Normal saline mice after 9 days of treatment. The scale bar is 200 μm (*P* < 0.001, n = 4). (b) Vital index of blood routine examination of normal saline/CNDs-treated group (*P* ＞ 0.05, n = 4).

To ensure the biosafety of CNDs using in animals repeatedly, we carried out the H&E-stained organ slices, in order to execute the histological examinations among normal saline group and the CNDs group (Figure 6a). Among the organs of CNDs treated group, no significant drug damages could be observed. Therefore, the damages of CNDs using in animals could be neglected. Furthermore, to verify the biosecurity of CNDs using in living animals, the healthy female BALB/c mice were injected with intravenous use of synthetic nanoparticles. After three days of injection, routine blood analysis was performed on the mice. The results of WBC, RBC, HGB, RDW, MCH and MCHC are vital data towards conditions of immune system of animals (Figure 6b). By comparing the normal saline group and the drug-treated group, we noticed that, the otherness of the four index between the normal saline group and the CNDs-treated group could be neglected (*P* ＞ 0.05, n = 4). Concluded the above experiments related with the safety tests of CNDs, CNDs are innoxious towards the immune system of mice.

## 4 Conclusions

To sum up, the eco-friendly cerium oxide nanodots (CNDs) we synthesized, were proved to have the ability of performing peroxidase-like activity in tumor cells while accomplishing catalase-like activity in normal cells. Proving that the CNDs themselves are capable of adjusting the ROS level according to the environmental differences of tumor cells and normal cells. In tumor cells surroundings, with acidic pH and high H_2_O_2_, CNDs accomplished the chemotherapeutic procedure via Fenton reaction, causing numerous ·OH rapidly. The nanoparticles catalyzed the decomposition of H_2_O_2,_ producing excessive·OH, whose highly toxicity could cause the death of tumor cells. On the other hand, while in the neutral and gentle environment, the CNDs maintained the biosafety after being applied in normal cells/tissues. Moreover, CNDs themselves also performed catalase-like activities. They exhibited the ability of promoting the elimination of reactive oxygen species, which even protect the cells from the irreversible damages on account of ROS. Besides, it is worth mentioning that not only did CNDs display conspicuous effect in vitro experiments, it also effectively restrained the growth of tumor while insuring the vital physical indicators of CNDs-treated mice remained in the normal level.

## Declarations

### Author contribution statement

Hui Wang: Conceived and designed the experiments; Performed the experiments; Analyzed and interpreted the data; Contributed reagents, materials, analysis tools or data; Wrote the paper.

Qi Wang: Performed the experiments; Analyzed and interpreted the data.

Jianyue Dong; Weiwei Jiang; Linghong Kong; Qiong Zhang: Performed the experiments.

Hanping Liu: Conceived and designed the experiments; Performed the experiments; Contributed reagents, materials, analysis tools or data.

### Funding statement

This study was supported by the 10.13039/501100001809National Natural Science Foundation of China [81671729] and the Science and Technology Program of Guangzhou [2019050001].

### Data availability statement

Data will be made available on request.

### Declaration of interest’s statement

The authors declare no conflict of interest.

### Additional information

No additional information is available for this paper.
